# The current landscape of imaging recommendations in cardiovascular clinical guidelines: toward an imaging-guided precision medicine

**DOI:** 10.1007/s11547-020-01286-9

**Published:** 2020-09-22

**Authors:** Antonio Esposito, Guglielmo Gallone, Anna Palmisano, Livia Marchitelli, Federica Catapano, Marco Francone

**Affiliations:** 1 Experimental Imaging Center, Radiology Unit, IRCCS Ospedale San Raffaele, Via Olgettina 60, 20132 Milan, Italy; 2 School of Medicine, Vita-Salute San Raffaele University, Via Olgettina 58, 20132 Milan, Italy; 3 Division of Cardiology, Department of Internal Medicine, Città della Salute e della Scienza, University of Turin, Turin, Italy; 4 Department of Radiological, Oncological and Pathological Sciences, Sapienza University of Rome, Viale del Policlinico 155, 00161 Rome, Italy

**Keywords:** AHA/ACC guidelines, ESC guidelines, Cardiovascular magnetic resonance, Cardiac computed tomography angiography

## Abstract

The purpose of this article is to provide an overview on the role of CT scan and MRI according to selected guidelines by the European Society of Cardiology (ESC) and the American College of Cardiology/American Heart Association (ACC/AHA). ESC and ACC/AHA guidelines were systematically reviewed for recommendations to CT and MRI use in specific cardiovascular (CV) clinical categories. All recommendations were collected in a dataset, including the class of recommendation, the level of evidence (LOE), the specific imaging technique, the clinical purpose of the recommendation and the recommending Society. Among the 43 included guidelines (ESC: *n* = 18, ACC/AHA: *n* = 25), 26 (60.4%) contained recommendations for CT scan or MRI (146 recommendations: 62 for CT and 84 for MRI). Class of recommendation IIa (32.9%) was the most represented, followed by I (28.1%), IIb (24%) and III (11.9%). MRI recommendations more frequently being of higher class (I: 36.9%, IIa: 29.8%, IIb: 21.4%, III: 11.9%) as compared to CT (I: 16.1%, IIa: 37.1%, IIb: 27.4%, III: 19.4%). Most of recommendation (55.5%) were based on expert opinion (LOE C). The use of cardiac CT and cardiac MR in the risk assessment, diagnosis, therapeutic and procedural planning is in continuous development, driven by an increasing need to evolve toward an imaging-guided precision medicine, combined with cost-effectiveness and healthcare sustainability. These developments must be accompanied by an increased availability of high-performance scanners in healthcare facilities and should emphasize the need of increasing the number of radiologists fully trained in cardiac imaging.

## Introduction

Non-invasive cardiovascular imaging with computed tomography (CT) and magnetic resonance imaging (MRI) has become integral part of the clinical routine, following the extraordinary technical evolution of the last 10–15 years.

Along with molecular and genomic studies, the recognized importance of cardiac imaging in early disease phenotyping, risk stratification and therapeutic guidance has brought to the development of an imaging-targeted precision medicine, which aims to change paradigms in various cardiological settings.

A crucial step of the process has been the translation of the clinical-radiological evidences into practice guidelines which allow to improve the value (quality and cost-effectiveness) of healthcare [1, 2]. Similarly, use of imaging-based appropriateness criteria has been shown to improve quality, reduce unnecessary imaging and lower healthcare costs [3].

Present article aimed to systematically provide an overview on the role of coronary CT angiography and cardiovascular magnetic resonance (CMR) in selected guidelines provided by the European Society of Cardiology (ESC) and of the American College of Cardiology/American Heart Association (ACC/AHA).

Our purpose would be to emphasize the primary importance of advance imaging in cardiovascular diseases, to promote its increasing utilization by the radiological community and possibly trigger scientific discussion regarding specific multimodality training, competency requirements and reimbursement policies by local governments.

## Guidelines review methods

To represent the current international landscape of guidance for the use of MRI and CT in cardiovascular medicine, we performed a systematic summary of recommendations regarding the use of imaging in selected guidelines of the ESC and of the ACC/AHA.

All the ESC-ACC/AHA guidelines relating to the following clinical categories were systematically reviewed for recommendations regarding the clinical use of MRI and CT: “Primary prevention,” “Ischemic Heart Disease,” “Valvular disease,” “Heart Failure,” “Pericardial disease,” “Arrhythmias” and “Hypertrophic Cardiomyopathy.” A full list of included guidelines along with the number of recommendations made in each document are reported in Table 1.

**Table 1:** ESC and ACC/AHA clinical guidelines included in the search, grouped by clinical category

ESC	PMID	No. of rec.	ACC/AHA	PMID	No. of rec.
*Primary prevention*					
Diabetes, Pre-Diabetes and Cardiovascular Diseases 2019	31497854	3	Primary Prevention 2019	30879355	1
Dyslipidaemias 2019	31591002	1	Blood Cholesterol 2018	30586774	2
Arterial Hypertension 2018	30165516	0	High Blood Pressure in Adults 2017	29133356	0
CVD Prevention in Clinical Practice 2016	27222591	1	Assessment of cardiovascular risk 2013	24222018	1
*Ischemic heart disease*					
Chronic Coronary Syndromes 2019	31504439	21	Primary PCI for STEMI (Focused Update) 2015	26498666	0
Myocardial Revascularization 2018	30165437	1	Non-ST-Elevation Acute Coronary Syndromes 2014	25249585	6
AMI in patients presenting with ST-Segment Elevation 2017	28886621	4	Stable Ischemic Heart Disease (focused update) 2014	25077860	0
ACS in patients presenting without persistent ST-Segment elevation 2015	26320110	2	Stable Ischemic Heart Disease 2012	23166211	30
			STEMI 2013	23247304	0
			Coronary Artery Bypass Graft Surgery 2011	22064599	0
			Percutaneous Coronary Intervention 2011	22064601	0
			Secondary Prevention for Atherosclerotic Vascular Disease 2011	22052934	0
*Valvular disease*					
Valvular Heart Disease 2017	28886619	1	Valvular Heart Disease (Focused Update) 2017	28298458	2
Infective endocarditis 2015	26590409	1	Infective endocarditis 2015	26373316	0
			Valvular Heart Disease 2014	24589853	11
*Heart failure*					
Acute and Chronic Heart Failure 2016	27206819	8	Heart Failure (Focused Update) 2017	28461007	0
			Heart Failure 2013	23741058	8
*Arrhythmia*					
Supraventricular Tachycardia 2019	31728553	0	Atrial Fibrillation (Focused Update) 2019	30703431	0
Syncope 2018	29562304	0	Bradycardia and Cardiac Conduction Delay 2018	30586772	7
Atrial Fibrillation 2016	27567408	0	Ventricular Arrhythmias and the Prevention of Sudden Cardiac Death 2017	29084731	7
Ventricular arrhythmias and the prevention of sudden cardiac death 2015	26320108	4	Syncope 2017	28280231	5
Cardiac pacing and cardiac resynchronization therapy 2013	23801822	0	Supraventricular Tachycardia 2015	26399663	0
			Atrial Fibrillation 2014	24685669	0
			Device-Based Therapy of Cardiac Rhythm Abnormalities 2008	18498951	0
*Pericardial disease*					
Pericardial diseases 2015	26320112	7			
*Hypertrophic cardiomyopathy*					
Hypertrophic Cardiomyopathy 2014	25173338	11	Hypertrophic Cardiomyopathy 2011	22068434	1

*ESC* European Society of Cardiology, *ACC* American College of Cardiology, *AHA* American Heart Association

**Fig. 1:**
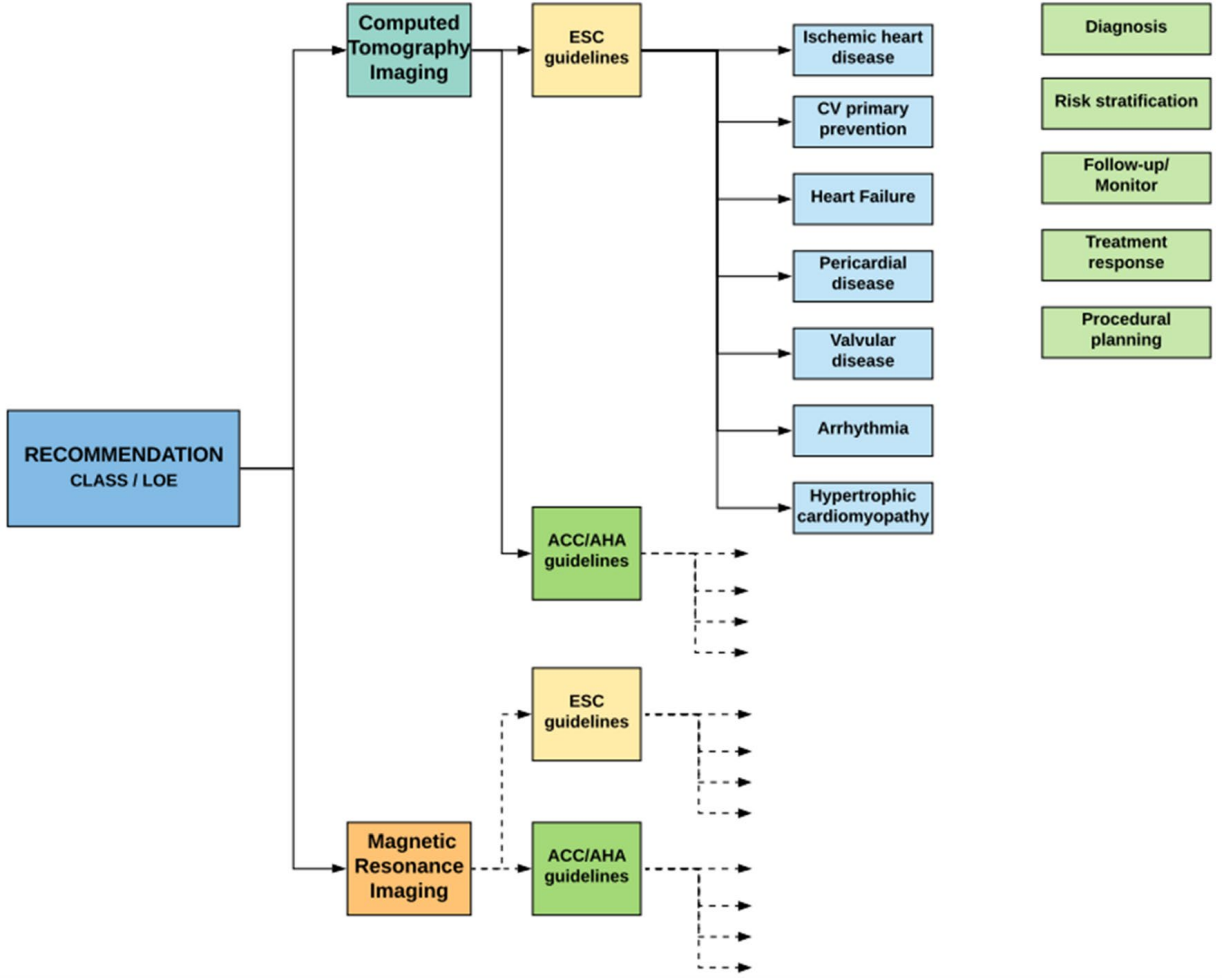
Guideline review process and classification. We systematically reviewed for recommendations regarding the imaging of cardiac structures, relating to specific clinical categories (cyan boxes). All the recommendations have been divided between the recommending society, and then both ESC and AHA/ACC recommendations have been classified among imaging technique (CT and MRI) and clinical purpose of the recommendation (light green boxes)

All recommendations were collected in a dataset, including the class of recommendation, the level of vidence (LOE) (Table 2), the specific imaging technique, the clinical purpose of the recommendation and the recommending society. A detailed flowchart of the review methodology and classification is reported in Fig. 1.

**Table 2:** Summary of predefined scales regarding "Classes of Recommendations" and "Level of Evidence" as adopted by the ESC guidelines. Only minor differences exist in the definitions of predefined scales adopted by the ACC/AHA guidelines

Classes of recommendations		Levels of evidence	
Class I	Evidence that the procedure is clinically useful	Level A	Data from multiple RCTs or meta-analysis
Class II	Conflicting evidence that the procedure is clinically useful	Level B	Data from a single RCT or large non-randomized studies
Class IIa	Weight of evidence in favor of efficacy		
Class IIb	Efficacy less well established	Level C	Expert consensus, data from small non-randomized studies
Class III	Evidence that the procedure is not clinically useful

Only recommendations regarding the imaging of cardiac and coronary structures were included in the present report. As a systematic review of all ESC and ACC/AHA clinical guidelines is beyond the scope of this report, some equally important cardiovascular imaging fields including vascular studies were not included in the search.

When the same recommendation was reported in more than one guideline from the same society, only the most recent was included. When a single recommendation regarded both MRI and CT imaging, it was double counted in order to allow separate analysis for both technologies. Statements regarding the use of CT and MRI imaging in the guideline document without an official specific recommendation were not included in the current analysis but are commented separately. Namely, research was expanded to consensus reports to comprehensively include the most relevant applications without formal recommendation (Paragraph 4).

## The current landscape of clinical cardiovascular guidelines

Of the 43 included guidelines (ESC: *n* = 18, ACC/AHA: *n* = 25), 26 (60.4%) contained recommendations regarding the clinical use of CT or MRI imaging (Table 1). Overall, 146 recommendations (ESC: *n* = 65, ACC/AHA: *n* = 81) were made, of which 62 for CT (ESC: *n* = 28, ACC/AHA : *n* = 37) and 84 for CMR (ESC: *n* = 37, ACC/AHA: *n* = 44) use.

### Class of recommendation

**Fig. 2:**
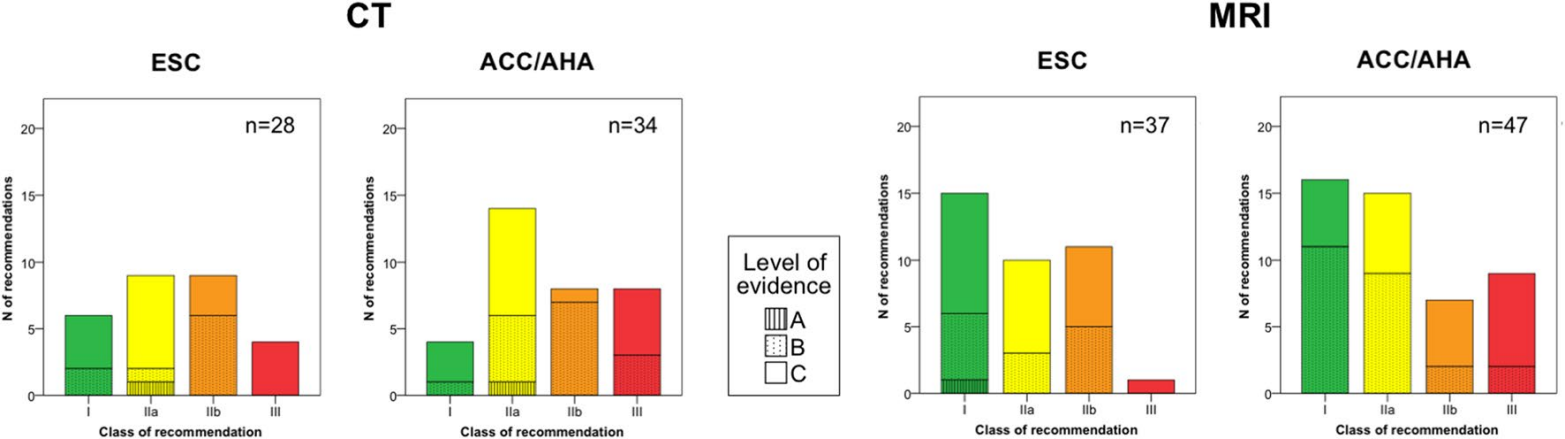
Recommendations for CT and MRI in ESC and ACC/AHA guidelines grouped by Class of recommendation and level of evidence. Histograms showing class of recommendation and level of evidence distribution of CT and MRI indications among ESC and ACC/AHA guidelines. Of notice, MRI recommendations are more frequently of higher class in both ESC and ACC/AHA guidelines

Overall, the most represented Class of recommendation was IIa (32.9%), followed by I (28.1%), IIb (24%) and III (11.9%). Different patterns were observed between MRI and CT, with MRI recommendations more frequently being of higher class (I: 36.9%, IIa: 29.8%, IIb: 21.4%, III: 11.9%) as compared to CT (I: 16.1%, IIa: 37.1%, IIb: 27.4%, III: 19.4%). Similar patterns were reported between ESC and ACC/AHA guidelines for both CT and MRI recommendations, except for class III MRI recommendations (“Not recommended”) which were more common among ACC/AHA vs. ESC guidelines (17% vs. 2.7%) (Fig. 2).

### Level of evidence

Regarding the evidence base to support imaging recommendations, most (55.5%) were based on expert opinion (LOE C), while only a small minority (2.1%) was based on evidence derived from adequately powered randomized controlled trials or high-quality meta-analyses (LOE A). This trend was similar for both CT (LOE A: 3.2%, LOE B: 40.3%, LOE C: 56.5%) and MRI (LOE A: 1.2%, LOE B: 44.0%, LOE C: 54.8%) recommendations, in both ESC and ACC/AHA guidelines (Fig. 2).

### Imaging techniques

**Fig. 3:**
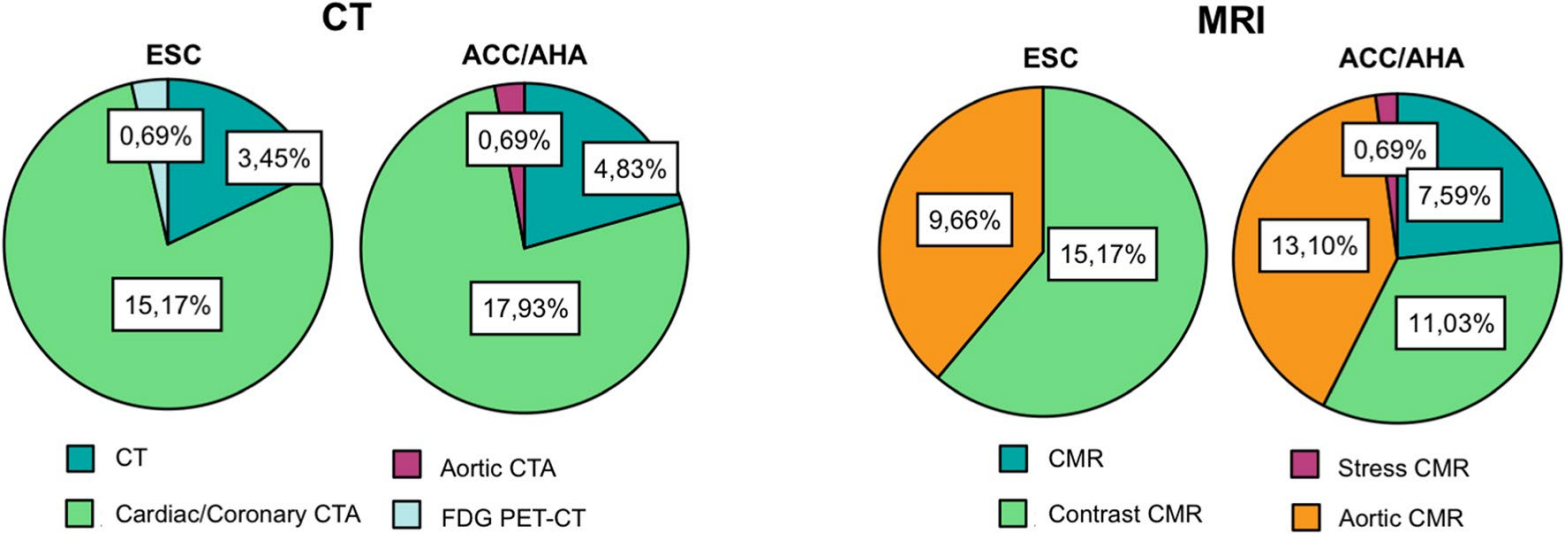
Rates of recommendations for CT and MRI by imaging technique in ESC and ACC/AHA guidelines. Pie charts on the left show the clear prevalence of recommendations rates of contrast-enhanced cardiac/coronary CT angiography among the other CT techniques in both ESC and ACC/AHA guidelines. Over MRI techniques (Pie Charts on the right), contrast-enhanced CMR is the most represented among recommendations, followed by stress CMR with similar rate

Of MRI techniques, contrast-enhanced CMR was the most represented among recommendations (45.8%), followed by stress CMR (39.8%). Among CT techniques, 77.4% recommendations related to contrast-enhanced cardiac/coronary CT angiography, followed by plain CT for the assessment of coronary artery calcium score (19.4%) (Fig. 3).

### Clinical categories

**Fig. 4:**
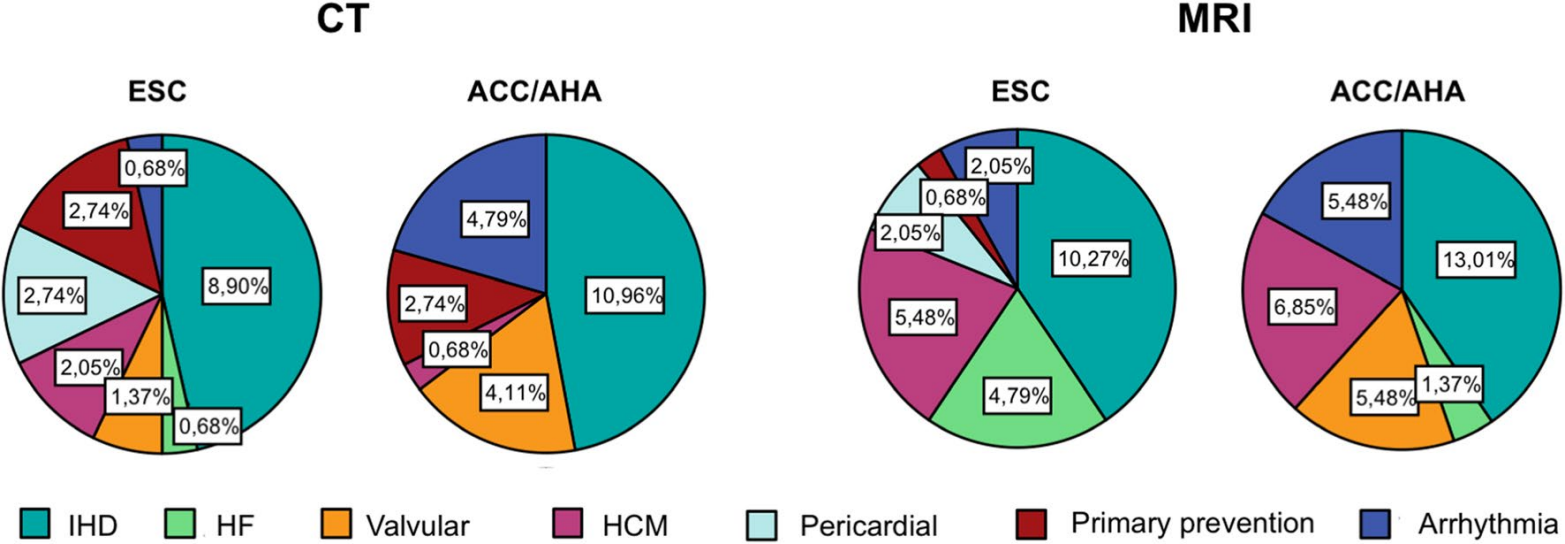
Rates of recommendations for CT and MRI by clinical category in ESC and ACC/AHA guidelines. Rates of recommendations for CT and MRI divided by clinical category, represented by Pie Charts, show the predominance of Ischemic heart disease among all of the others, with similar patterns between ESC and ACC/AHA guidelines

Ischemic heart disease was the most represented clinical category among recommendations (43.2%), for both MRI (40.5%) and CT (46.8%), with similar patterns between ESC and ACC/AHA guidelines. Of these, 63.6% recommendations referred to the chronic and 36.4% to the acute coronary syndromes setting. The distribution of recommendations among other clinical indications is detailed in Fig. 4.

### Scope of the recommendation

“Diagnosis” was the most common clinical purpose of imaging recommendations (61.0%), for both CT (61.3%) and MRI (60.7%), in both ESC and ACC/AHA guidelines. This was followed by “risk stratification” (23.3%), for both CT (24.2%) and MRI (22.6%), in both ESC and ACC/AHA guidelines.

**Fig. 5:**
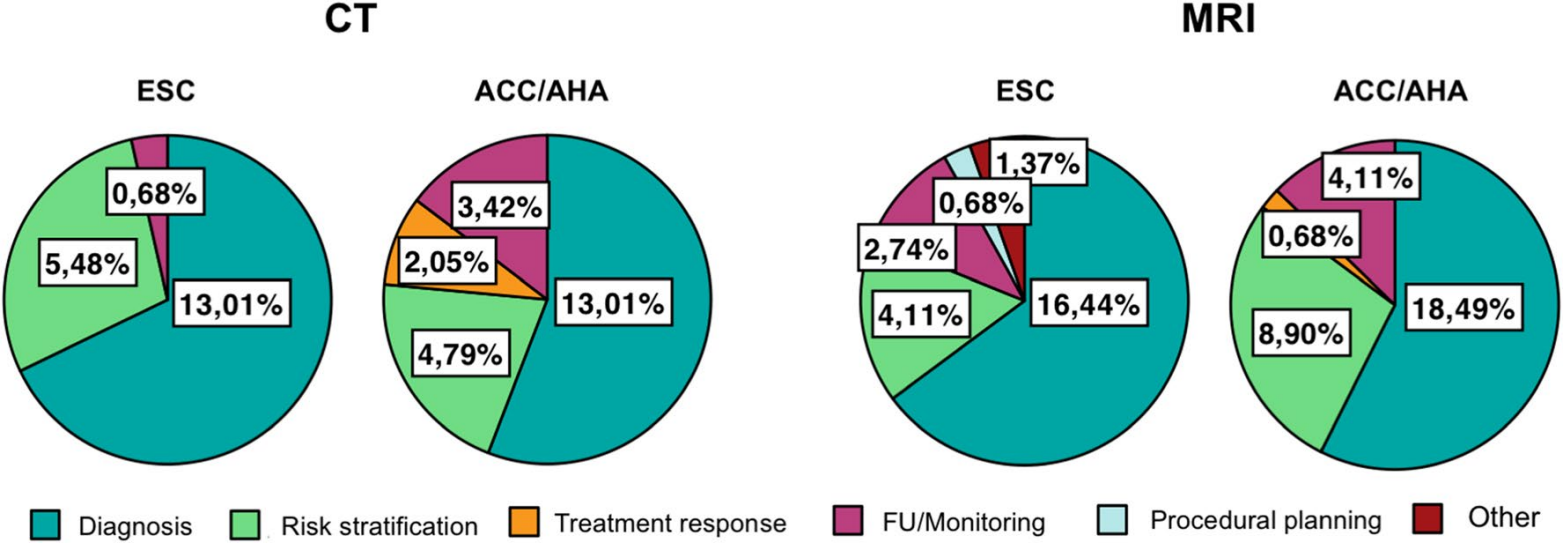
Rates of recommendations for CT and MRI by indication purpose in ESC and ACC/AHA guidelines. Scope of the recommendation, illustrated by Pie Charts, defines CT and MRI as crucial tools in the initial clinical workup of patients, for both diagnosis and risk stratification. Of notice, a single recommendation is addressed to preprocedural planning, highlighting a huge gap between guidelines and clinical practice

Among all the included documents, a single recommendation from the ESC guidelines for hypertrophic cardiomyopathy was related to preprocedural planning (“CMR with LGE may be considered before septal alcohol ablation or myectomy, to assess the extent and distribution of hypertrophy and myocardial fibrosis”) [4] (Fig. 5).

## Recommendations bottom line: specific settings of clinical use

### Primary prevention

Current recommendations in this setting concern detection of subclinical coronary atherosclerosis and detection of obstructive/ischemia-causing coronary atherosclerosis among at risk asymptomatic individuals.

Regarding the former goal, coronary artery calcium (CAC) is a highly specific marker of atherosclerotic burden [5], able to improve atherosclerotic cardiovascular disease event prediction among asymptomatic individuals over traditional risk factors [6–9]. The approach of ESC and ACC/AHA guidelines to CAC score diverges, with ESC guidelines expressing weak recommendations for CAC as a risk modifier among low-to-intermediate risk individuals based on clinical assessment (IIb, B). ACC/AHA conversely embraces a broader use of CAC as a potential tool to refine lipid-lowering therapy allocation among patients with uncertain predicted benefit from treatment initiation (IIa, B).

Regarding the detection of obstructive disease among asymptomatic individuals, no recommendations are expressed by ACC/AHA guidelines, while a weak (IIb, B) recommendation for the use of either coronary CT angiography or functional tests (including stress CMR) in patients with diabetes, strong family history or high clinical risk is made by ESC guidelines. The use of coronary CT angiography or functional imaging in patients not fulfilling these requirements is contraindicated (III, C).

### Ischemic heart disease

In the setting of chronic coronary syndromes (CCS), 2019 ESC guidelines recommend either coronary CT angiography or stress CMR or their combined use to diagnose and to risk stratify obstructive disease among symptomatic patients based on their pre-test disease probability, local expertise and availability, and anticipated quality of the exam (I to IIa, B to C) [10]. Less emphasis on coronary CT angiography use for CCS diagnosis is posed by the outdated 2012 ACC/AHA guidelines, which overall favors functional imaging tests as the exam of choice [11].

Among patients with a prior CCS diagnosis who needs risk assessment (new/worsening symptoms or high clinical prognostic risk), ESC guidelines highlight functional imaging tests as the exam of choice (I, B). Conversely, according to the ACC/AHA guidelines also coronary CT angiography may be considered in this setting (IIa to IIb, B to C). Of note, ACC/AHA guidelines formally contemplate the use of coronary CT angiography for the assessment of patency of bypass grafts or of coronary stents ≥ 3 mm in diameter in patients with new/worsening symptoms (IIb, B); a similar statement is made by ESC guidelines without a formal recommendation.

In the setting of non-ST-segment elevation acute coronary syndromes, ESC guidelines recommend stress imaging to look for inducible ischemia before coronary angiography among patients with suspected low-risk unstable angina (I, A). For both ESC and ACC/AHA guidelines, among patients without prior history of coronary artery disease presenting with chest pain and having an inconclusive diagnostic assessment, coronary CT angiography should be considered as an alternative to coronary angiography (IIa, A) [12, 13].

According to ESC guidelines, among patients with ST-elevation myocardial infarction, stress CMR may be performed following primary percutaneous coronary intervention (PCI) to assess residual ischemia and viability (IIb, C). Furthermore, CMR should be considered when echocardiography is suboptimal, both in-hospital (after primary PCI) and after discharge, for the quantification of left ventricular function (IIa, C) [14]. No recommendations in this setting are provided by the outdated 2013 ACC/AHA guidelines [15].

### Valvular disease

Echocardiography plays a leading role in the study of valvular disease, according to both ESC and ACC/AHA guidelines [16, 17].

In this setting, current ACC/AHA guidelines indicate CT and CMR as second-level examination in patients with a poor acoustic window that does not allow the correct evaluation of valve function and/or morphology.

ESC guidelines do not provide a specific class of recommendations for the use of CT and CMR, although their potential clinical value is acknowledged in the documents. Similarly, ESC guidelines on infective endocarditis do not contain formal recommendation for CT or CMR, although CT findings are listed in the modified Duke diagnostic criteria [18]. On the other hand, ACC/AHA guidelines on infective endocarditis suggest the use of CT in patient with suspected valve thrombosis to asses leaflets function and the extent of the thrombus.

Both ESC and ACC/AHA guidelines suggest coronary CT angiography, as alternative to invasive angiography, in order to exclude coronary artery disease (CAD) in low-risk patients candidate to valve surgery (ESC IIa LOE C; ACCF/AHA IIa LOE B) [16, 17].

### Heart failure

In the evaluation of heart failure, both ESC and ACC/AHA guidelines suggest the use of CMR in case of inadequate echocardiography findings or suspected infiltrative process and/or when scar burden need to be assessed [19, 20].

CMR has a Class I recommendation (LOE C) to identify myocarditis in patients with new onset or established heart failure according to ESC guidelines [19].

Coronary CT angiography can be used to rule out obstructive CAD in patients with low-to-intermediate pre-test probability and equivocal non-invasive stress tests, as recommended by ESC guidelines, with moderate appropriateness and level of evidence (IIb LOE C) [19].

### Arrhythmias

In the widespread setting of cardiac arrhythmias, most of CT and CMR indications, in both ESC and ACC/AHA guidelines, regard ventricular arrhythmias and prevention of sudden cardiac death.

In particular, CT and CMR are recommended in selected patients in whom structural heart disease or myocardial infiltrative processes are suspected [21, 22].

Moreover, ACC/AHA guidelines suggest CT and/or CMR examinations if structural heart disease is suspected, yet not confirmed by other diagnostic modalities, in selected patients with bradycardia or bundle branch block [23] and in suspected cardiac syncope [24].

### Pericardial disease

Notably, no dedicated ACC/AHA guidelines are available for pericardial disease. On the other hand, 2015 ESC pericardial disease guidelines strongly recommend CT and/or CMR as second-level testing for diagnostic workup in pericarditis (I LOE C), with a special mention in the diagnosis of constrictive pericarditis (I LOE C). Weaker recommendations are provided for pericardial effusion (IIa LOE C) [25].

### Hypertrophic cardiomyopathy

In the workup of patients with known or suspected hypertrophic cardiomyopathy (HCM), both ESC and ACC/AHA guidelines emphasize the role of CMR [4, 20, 26]. In particular, CMR is suggested as diagnostic tool in case of inadequate echocardiographic windows (I LOE B) and to exclude infiltrative processes (ESC IIa LOE B; ACC/AHA IIb LOE C) [4]. In patients with confirmed diagnosis of HCM, CMR can be useful to assess cardiac anatomy, ventricular function, and the extent of myocardial fibrosis, with special mention for evaluation of apical hypertrophy and aneurysm (ESC IIa LOE C; ACC/AHA IIa LOE B) [4].

Moreover, ACC/AHA suggests that CMR can provide additional information in prognostic stratification and/or therapeutic planning [20].

On the other hand, ESC recommends CMR to assess the extent and distribution of hypertrophy and myocardial fibrosis prior to septal alcohol ablation or myectomy (IIb LOE C) and suggests its role as follow-up technique in stable patients (IIb LOE C) [4].

In this scenario, the use of CT angiography is indicated to assess for possible concomitant CAD in low-/intermediate-risk patients according to both ESC and ACC/AHA guidelines (IIa LOE C) and in the diagnosis of HCM, when CMR is contraindicated according to ESC (IIa LOE C) [4, 20].

## Statements on clinical use of cardiovascular imaging without formal recommendations

Over the last years, an impressive technological development involved CT and CMR, improving their performances and application fields. This advancement was associated with a relatively recent expansion of the possible clinical applications, including restrictive cardiomyopathy, inflammatory and arrhythmogenic cardiomyopathy and structural intervention. The application of CT and CMR in these settings is still not included in the aforementioned guidelines, but in some cases is part of the clinical routine and is supported by appropriateness criteria suggested by dedicated expert consensus documents, as reported below.

### Restrictive cardiomyopathy

According to a recent expert consensus document from European Association of Cardiovascular Imaging (EACVI) and the Working Group on Myocardial and Pericardial diseases of the ESC [27], CMR is recommended for the diagnosis of restrictive cardiomyopathy thorough an accurate assessment of cardiac chambers volume and mass, beside myocardial tissue characterization.

No formal recommendations have been reported about the use of mapping in this setting, although the clinical experience and the most recent literature suggest that tissue relaxometry imaging, particularly with T1 and T2* mapping, may play a fundamental role in infiltrative cardiomyopathies and iron overload, respectively.

Moreover, when echocardiography is nondiagnostic and CMR contraindicated, CT can be adopted for the evaluation of cardiac chambers volume, myocardial mass, myocardial scar and extracellular volume fraction (ECV) quantification.

### Inflammatory cardiomyopathies

According to the recent expert consensus document on inflammatory nonischemic cardiomyopathy [28], CMR may be considered as a first-line diagnostic tool for diagnostic workup of acute myocardial inflammation. The use of mapping to detect myocardial inflammation is strongly suggested in this setting, because it has a positive impact on diagnostic accuracy [29] that could be further improved through a mapping-based assessment of myocardial hyperemia [30]. Similarly, CMR may be useful for the identification of various chronic inflammatory conditions, ranging from chronic myocarditis to sarcoidosis to human immunodeficiency virus disease, and to exclude inflammatory substrate of new onset arrhythmias [31]. As previously indicated CMR has a role in identifying myocardial inflammation in patients with new onset or established heart failure [19].

### Arrhythmogenic cardiomyopathy

According to the recent expert consensus of the EACVI [32], CMR is indicated to support the diagnosis of arrhythmogenic cardiomyopathy together with ECG, histological and functional evaluation, both in the early and advanced disease.

Because of its progressive nature, repeated cardiac imaging is needed to follow disease progression and for risk assessment of life-threatening ventricular arrhythmias.

### Transcatheter procedure and structural interventions

Over the last years, the field of transcatheter valve and structural interventions has experienced a huge expansion supported by enormous technical development. Currently, ACC/AHA and ESC guidelines on this issue are not available. Recent expert consensus documents, from both cardiological and radiological societies [33, 34], recommend CT for procedural planning in the setting of transcatheter aortic valve replacement (TAVR) and valve-in-valve procedures.

CT is widely used for procedural planning (vascular accesses, annular sizing, determination of risk of annular injury and coronary occlusion, fluoroscopic angle prediction) and patients’ selection. Similarly, a recent expert consensus [35] stated the importance of CT in procedural planning of transcatheter mitral valve replacement, and in the transcatheter closure of paravalvular leakage, atrial septal defect, left atrial appendage and oval foramen. There is no consensus regarding the role of CT after TAVR, although CT may adjuvate the diagnosis in case of valve thrombosis, infective endocarditis or structural degeneration.

## Final remarks

The ESC and AHA/ACC guidelines convey few general remarks:

1. CMR is generally considered appropriate when myocardial tissue characterization (scar, infiltrative disease, inflammation, etc.) is pivotal for the diagnosis and when echocardiography fails to provide an accurate morpho-functional assessment;
2. Coronary CT angiography gained a central role in the setting of chronic coronary syndrome (i.e., stable angina) according to the recently updated 2019 ESC guidelines. A limited role to the method is conversely conferred by the ACC/AHA guidelines, likely because of the non-up-to-date version of the latest documents (2012–2014).
3. Only a small minority of the recommendations about the use of CT and CMR in the ESC and ACC/AHA guidelines are supported from adequately powered randomized controlled trials or high-quality meta-analyses (LOE A) (Fig. 2). This observation highlights the urgent need of financial supports to large randomized controlled trials, in order to obtain unbiased results supporting the clinical value of cardiac CT and CMR in real clinical world.

Beyond these main messages, several additional considerations could be raised.

The number of class I recommendations is significantly higher for CMR than coronary CT angiography (Fig. 2), likely reflecting the younger age of coronary CT and a more established attitude of cardiologists to use a nonionizing “echocardiography-like” imaging method. This scenario is likely to change in the next future, as a consequence of the development and standardization of new promising CT-derived techniques, such as CT perfusion [36], CT delayed enhancement [37, 38], CT-based extracellular volume quantification [39] and CT-based fractional flow reserve [40].

Although CMR has several formal recommendations in both ESC and ACC/AHA guidelines, the role of parametric mapping techniques is still not established. This could be surprising considering the growing evidences about the positive impact of mapping in the diagnostic and prognostic evaluation of several conditions, as T2* mapping for iron overload, T1 mapping in Fabry disease, ECV in cardiac amyloidosis and parametric mapping in myocarditis [41, 42]. Nevertheless, T2* mapping is currently recommended in disease-specific consensus statement of experts [43], as well as both T1 and T2 mapping technique in expert consensus about the use of CMR in inflammatory myocardial diseases [28]. Despite the Society of Cardiovascular Magnetic Resonance provides clinical recommendation for the use of mapping in this field [44], the inclusion of this technique in the official guidelines of the main cardiological societies is still needed, also with the aim of encouraging a wide technological and cultural upgrading.

Surprisingly, there is a limited consideration for stress CMR as a tool for detecting ischemia in the ACC/AHA guidelines (Fig. 3). This could be shocking, considering that the diagnostic accuracy of stress CMR to detect significant coronary artery disease and to guide revascularization has been proved also by controlled randomized trails [45, 46]. Moreover, the prognostic value and cost-effectiveness of stress CMR were investigated with good results [47]. On the other hand, the ESC guidelines [18] recognize the role of CMR to detect myocardial ischemia, equally to nuclear medicine, but more investments are probably needed in the next future to increase the availability and the technological level of the MR scanners.

Although endovascular or mini-invasive preprocedural planning represent one of the most rapidly evolving application of three-dimensional cardiac imaging techniques, particularly in the settings of structural heart interventions and arrhythmias ablation, formal recommendations provided by the major cardiological society are very limited (Fig. 5), due to the lack of large randomized trial confirming their value. However, several evidences and specific consensus statements have defined CT and CMR as useful tools to support extra-coronary heart interventions [33, 34, 48, 49].

In conclusion, the use of cardiac CT and cardiac MR in the risk assessment, diagnosis, therapeutic and procedural planning is in continuous development, driven by an increasing need to evolve toward an imaging-guided precision medicine, combined with cost-effectiveness and healthcare sustainability. A clear sign of this evolution is the new role recently recognized to the coronary CT angiography in the 2019 ESC Guidelines on Chronic Coronary Syndromes, as recommended initial test for patients with a low to moderate clinical likelihood of disease. However, these developments must be accompanied by an increased availability of high-performance scanners in healthcare facilities and should emphasize the need of increasing the number of radiologists fully trained in cardiac imaging, exploiting and reinforcing different solutions, including the exchanging programs for residents, the education programs of the national and international scientific societies, as well as the dedicated master classes.

## Data Availability

‘Not applicable’ for that section. ‘Not applicable’ for that section.
